# Supplementary material for: TransST: transfer learning embedded spatial factor modeling of spatial transcriptomics data
Source: BMC Bioinformatics. 2025 Nov 6;26:274. doi: 10.1186/s12859-025-06099-z (PMC12593783; doi:10.1186/s12859-025-06099-z)
Supplement: Supplementary file 1 [file 12859_2025_6099_MOESM1_ESM.pdf]

Supplementary Material for TransST

1 Selection of number of clusters K

Identifying the suitable number of clusters is a pivotal stage in clustering analysis, significantly influencing the accuracy and meaningful interpretation of outcomes. The quest for the optimal number of clusters unveils inherent patterns and relationships within the data, assisting in the recognition of distinct groups or categories. This not only enriches comprehension of the data but also streamlines targeted decision-making processes.

Statistical models employing likelihood-based methods, such as Bayesian Information Criterion (BIC), serve as invaluable tools in the determination of the number of clusters. Likelihood-based approaches offer an objective and data-driven methodology for cluster selection, enhancing the precision and reliability of the clustering process. Instead of using the classical BIC formulation, which is only valid when the sample size is much larger than the number of parameters (Giraud, 2021), we adopt the high-dimensional BIC (HBIC) (Wang et al., 2013) for estimating K. More specifically, we define the HBIC by

$$\text{HBIC} = -2\ell + p^* \log[\log(n) + p^*],$$

where ℓ is the loglikelihood, $p^* = k(q+q(q+1)/2)+1$ denotes the number of parameters to be estimated, and k is the candidate of the number of clusters.

2 Simulation

Throughout the simulation studies, we consider $K = 4$, $q = 10$, $p = 1000$, $n_1 = 1875$, and $n_0 = 625$. The whole simulation procedure follows DR.SC (Liu et al., 2022). To simulate the cluster labels z_i by the spatial coordinates, we first simulate a rectangular lattice from a K-state Potts model, implemented by the function sampler.mrf in the R package *GiRaF*. Next, we generate the low-dimensional representation $\mathbf{u}_i$ and $\mathbf{v}_i$ from a multivariate normal distribution $\mathcal{N}(\boldsymbol{\mu}_k, \boldsymbol{\Sigma}_k)$. For the mean, we let $\boldsymbol{\mu}_1 = [5, 0, \cdots, 0]$, $\boldsymbol{\mu}_2 = [0, 5, 0, \cdots, 0]$, $\boldsymbol{\mu}_3 = [0, 0, 5, 0, \cdots, 0]$, and $\boldsymbol{\mu}_4 = [0, 0, 5, \cdots, 5]$. For the variance-covariance matrix, we generate a diagonal matrix $\boldsymbol{\Sigma}_k$ whose diagonal elements are all 1 if $k > K/2$ and the other elements follow a uniform distribution $\mathcal{U}(0.1, 10)$ if $k \leq K/2$. A random noise matrix $\boldsymbol{\eta}\boldsymbol{\eta}^\top$, where $\eta_i \sim \mathcal{N}(0, 0.1^2)$, is further added to $\boldsymbol{\Sigma}_k$. To simulate the differentially expressed genes, we let $\mathbf{W} = [\mathbf{I}_{q\times q}, \mathbf{0}_{q\times(p-q)}]^\top$ and a noise from $\mathcal{U}(-0.1, 0.1)$ is further added. With this generating procedure, the four clusters in $\mathbf{X}_{tgt}$ are differentiated by genes 1, 2, 3, and 4–10, respectively. Finally, we simulate the high-dimensional gene expression vectors $\mathbf{x}_{src,i} \sim \mathcal{N}(\mathbf{W}\mathbf{u}_i, \boldsymbol{\Lambda})$ and $\mathbf{x}_{tgt,i} \sim \mathcal{N}(\mathbf{W}\mathbf{v}_i, \boldsymbol{\Lambda})$, where the diagonal element λ_j in $\boldsymbol{\Lambda}$ follows a uniform distribution $\mathcal{U}(0.1, 2)$.

3 Real data preprocessing

HER-2 positive tumors: We first remove cells if their cluster sizes are less than 20 or they are labelled as "undetermined". More information about the sizes of the eight samples after step 1 are included in the supplementary materials. Second, we log-normalize the gene-expression matrix with a library size. Third, we keep the genes which are shared by all the eight samples. Finally, for each target sample, we choose the top $p = 2000$ genes, sorted by their variances.

Mouse embryo: We preprocess the dataset by the following step. Log-normalization is applied to the gene-expression matrix with a library size for each

sample. No gene selection is performed since the dimensionality $p = 351$ is relatively small.

cSCC: We first take the log-normalization on the target data with library size and then select the top $p = 2000$ genes, sorted by their variances.

4 Implementation of TransST

To simplify notations, we use the same $\boldsymbol{\theta}$ to denote the parameter space.

pLDR: To estimate the parameters $\boldsymbol{\theta} = (\{\boldsymbol{\mu}_k, \boldsymbol{\Sigma}_k\}_{k=1}^K, \boldsymbol{\Lambda}, \mathbf{W})$, we maximize the observed likelihood, which is given by

$$\begin{aligned} L(\boldsymbol{\theta}) &= \prod_{i=1}^{n_1} f(\mathbf{x}_{src,i}|z_i) = \prod_{i=1}^{n_1} \int_{\mathbf{u}_i} f(\mathbf{x}_{src,i}, \mathbf{u}_i|z_i) d\mathbf{u}_i \\ &= \prod_{i=1}^{n_1} \int_{\mathbf{u}_i} f(\mathbf{x}_{src,i}|\mathbf{u}_i) f(\mathbf{u}_i|z_i) d\mathbf{u}_i \\ &= \prod_{i=1}^{n_1} \prod_{k=1}^{K} I\{z_i = k\}(2\pi)^{-p/2}|\mathbf{S}_k|^{1/2} \exp\left[-\frac{1}{2}\left(\mathbf{u}_i - \mathbf{W}\boldsymbol{\mu}_k\right)^\top \mathbf{S}_k \left(\mathbf{u}_i - \mathbf{W}\boldsymbol{\mu}_k\right)\right], \end{aligned}$$

where $\mathbf{S}_k = \boldsymbol{\Lambda}^{-1} - \boldsymbol{\Lambda}^{-1}\mathbf{W}\mathbf{C}_k^{-1}\mathbf{W}^\top\boldsymbol{\Lambda}^{-1}$ and $\mathbf{C}_k = \mathbf{W}^\top\boldsymbol{\Lambda}^{-1}\mathbf{W} + \boldsymbol{\Sigma}_k^{-1}$. Note that the conditional independence $\mathbf{x}_{src,i} \perp\!\!\!\perp z_i|\mathbf{u}_i$ is required, which is mild and frequently used in the literature of sufficient dimension reduction.

spGMM: To estimate the cluster labels and the model parameters, we use the EM algorithm based on iterative conditional model (ICM) (Besag, 1986). The ICM step estimate the cluster labels by maximizing the posterior distribution

$$f(z_i|\mathbf{v}_i, z_{N_i}) \propto f(\mathbf{v}_i|z_i) f(z_i|z_{N_i}).$$

Therefore, maximizing the posterior is equivalent to minimizing

$$\hat{z}_i = \arg\min_{z_i} \ell_1(z_i), \tag{1}$$

where

$$\ell_1(z_i) = \sum_{k=1}^{K} \left\{ \frac{1}{2}\log|\mathbf{\Sigma}_k| + \frac{1}{2}(\mathbf{v}_i - \boldsymbol{\mu}_k)^\top \mathbf{\Sigma}_k^{-1}(\mathbf{v}_i - \boldsymbol{\mu}_k) + \beta \sum_{i' \in N_i} (1 - I\{z_i = z_{i'}\}) \right\}.$$

Furthermore, the pseudo observed loglikelihood is given by

$$\ell = \log f(\mathbf{V}|\hat{\mathbf{z}}) = \sum_{i=1}^{n_0} \log \sum_{k=1}^{K} f(\mathbf{v}_i|z_i = k) f(z_i = k|\mathbf{z} = \hat{\mathbf{z}}). \tag{2}$$

E-step: Next, we derive the lower bound of the pseudo observed loglikelihood by

$$\begin{aligned}
\log f(\mathbf{v}) &= \log \int_z f(\mathbf{v}, z) dz \\
=& \sum_{i=1}^{n_0} \log \int_{z_i} f(\mathbf{v}_i|z_i) f(z_i|z_{N_i}) dz_i \\
=& \sum_{i=1}^{n_0} \log \int_{z_i} f(z_i|\mathbf{v}_i, z_{N_i}) \frac{f(\mathbf{v}_i|z_i) f(z_i|z_{N_i})}{f(z_i|\mathbf{v}_i, z_{N_i})} dz_i \\
\geq& \sum_{i=1}^{n_0} E_{\boldsymbol{\theta}^{(t)}} \log(f(\mathbf{v}_i|z_i) f(z_i|z_{N_i})) \\
=& Q(\boldsymbol{\theta}; \boldsymbol{\theta}^{(t)}) - \sum_{i=1}^{n_0} E_{\boldsymbol{\theta}^{(t)}} \log f(z_i|\mathbf{v}_i, z_{N_i}),
\end{aligned}$$

where $\boldsymbol{\theta} = (\{\boldsymbol{\mu}_k, \mathbf{\Sigma}_k\}_{k=1}^{K}, \beta)$ and the inequality is due to the Jensen's inequality. Notice that

$$\begin{aligned}
Q(\boldsymbol{\theta}; \boldsymbol{\theta}^{(t)}) &= \sum_{i=1}^{n_0} \sum_{k=1}^{K} R_{ik}^{(t)} [\log f(\mathbf{v}_i|z_i = k) + \log f(z_i = k|z_{N_i})] \\
&\propto \sum_{i=1}^{n_0} I_{i1} + I_{i2},
\end{aligned}$$

where

$$I_{i1} = \sum_k R_{ik}^{(t)} \left\{ -\frac{1}{2} \log |\boldsymbol{\Sigma}_k| - \frac{1}{2} (\mathbf{v}_i - \boldsymbol{\mu}_k)^\top \boldsymbol{\Sigma}_k^{-1} (\mathbf{v}_i - \boldsymbol{\mu}_k) \right\},$$

$$I_{i2} = -\sum_k R_{ik}^{(t)} \left\{ \beta \sum_{i' \in N_i} (1 - I\{z_i = z_{i'}\}) \right\},$$

and the posterior probability of cell i belonging to cluster k is

$$R_{ik} = P(z_i | \mathbf{v}_i, z_{N_i}) = \frac{P(\mathbf{v}_i | z_i = k) P(z_i = k | z_{N_i})}{\sum_{k'} P(\mathbf{v}_i | z_i = k') P(z_i = k' | z_{N_i})}.$$

M-step: Taking the derivative of $Q(\boldsymbol{\theta}; \boldsymbol{\theta}^{(t)})$ with respect to $\boldsymbol{\theta}$, we have

$$\hat{\boldsymbol{\mu}}_k = \sum_{i=1}^{n_0} R_{ik}^{(t)} \mathbf{v}_i / \sum_{i=1}^{n_0} R_{ik}^{(t)} \tag{3}$$

$$\hat{\boldsymbol{\Sigma}}_k = \sum_{i=1}^{n_0} R_{ik}^{(t)} (\mathbf{v}_i - \boldsymbol{\mu}_k)(\mathbf{v}_i - \boldsymbol{\mu}_k)^\top / \sum_{i=1}^{n_0} R_{ik}^{(t)}. \tag{4}$$

To estimate β, we use the line search method, i.e., $\hat{\beta} = \arg\max_\beta \sum_i I_{i2}$ for $\beta \in [0, 1]$.

5 Human dorsolateral prefrontal cortex datasets

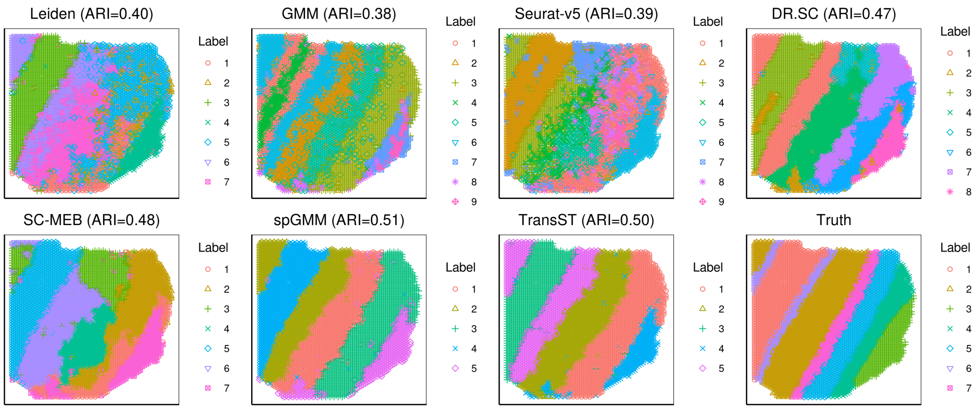

Fig. 1 Spatial heatmap for Sample 151507.

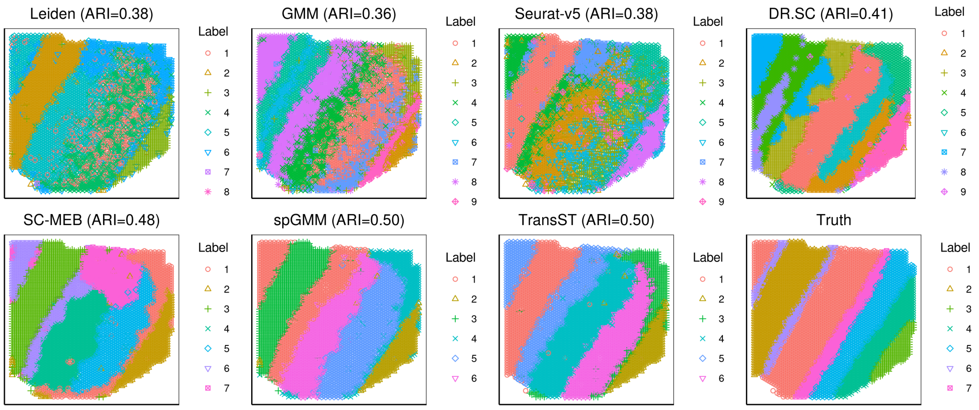

Fig. 2 Spatial heatmap for Sample 151508.

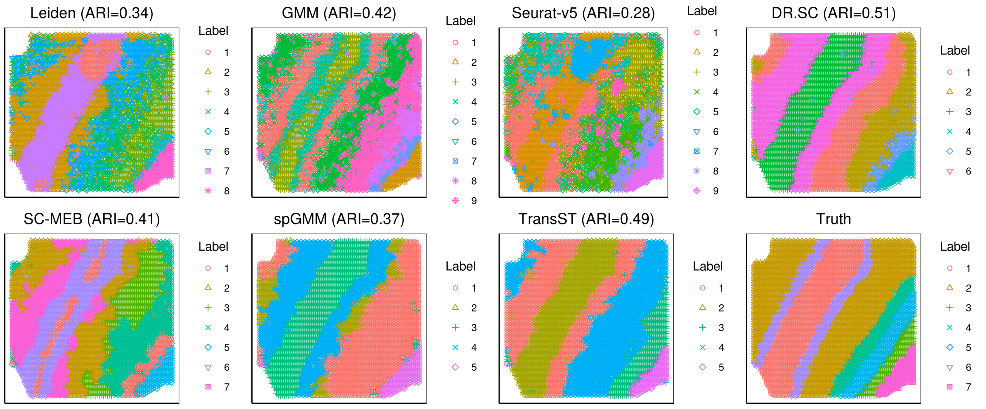

Fig. 3 Spatial heatmap for Sample 151509.

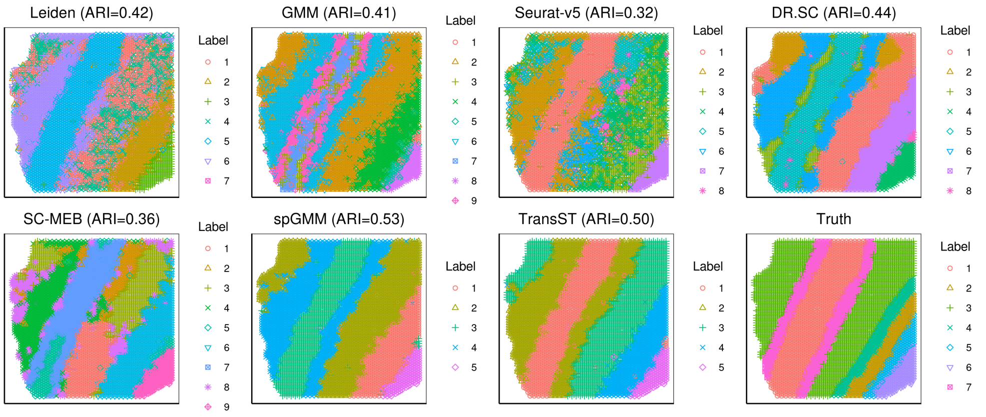

Fig. 4 Spatial heatmap for Sample 151510.

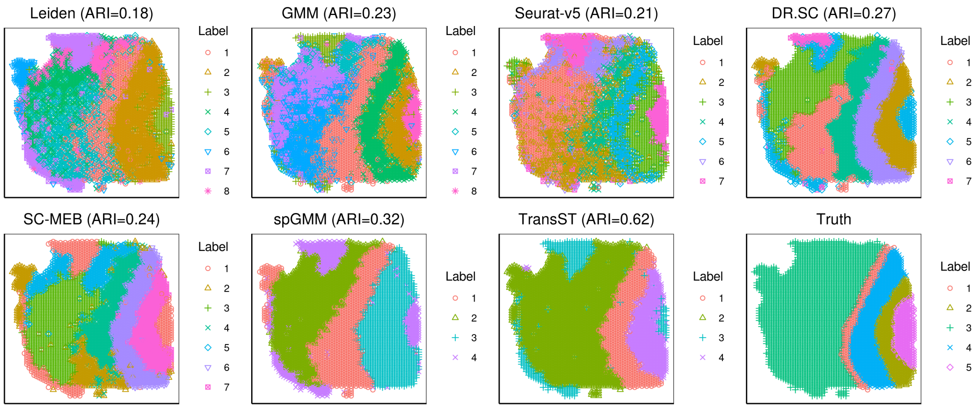

Fig. 5 Spatial heatmap for Sample 151670.

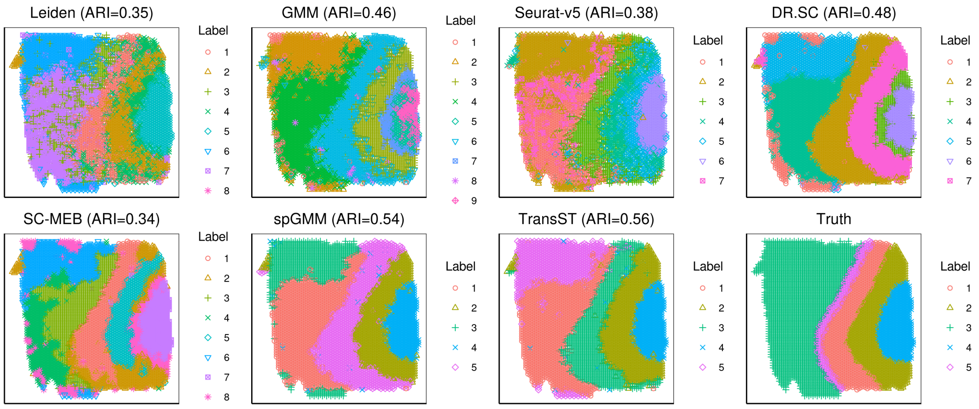

Fig. 6 Spatial heatmap for Sample 151671.

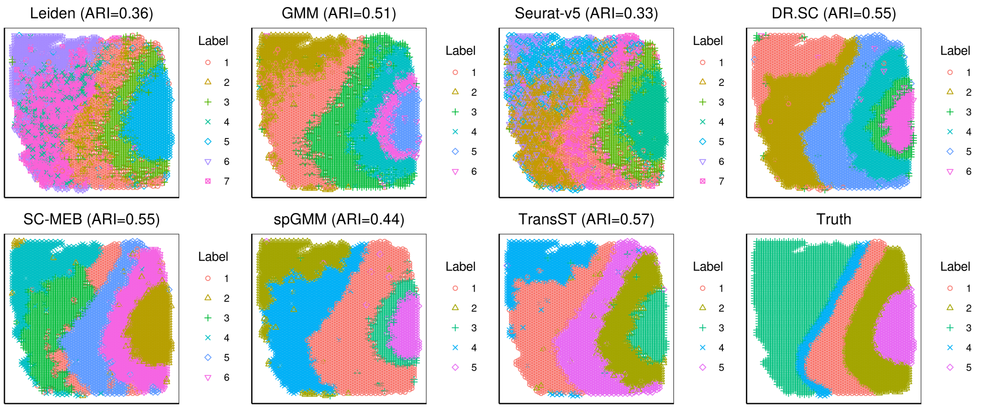

Fig. 7 Spatial heatmap for Sample 151672.

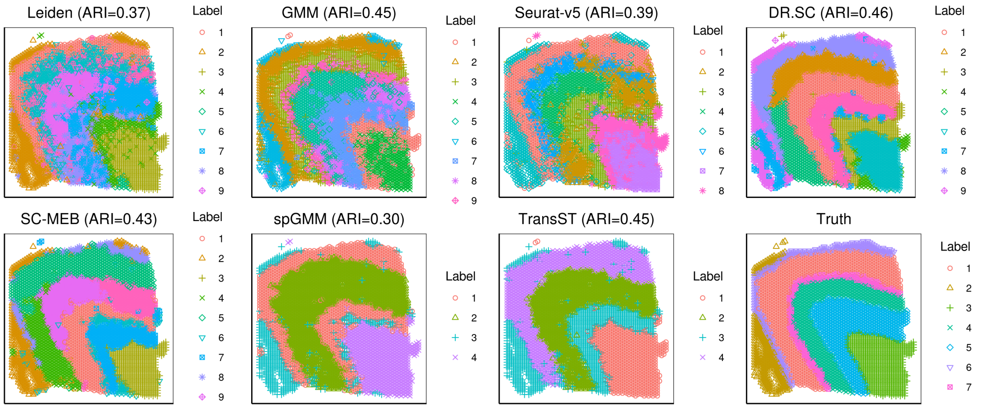

Fig. 8 Spatial heatmap for Sample 151673.

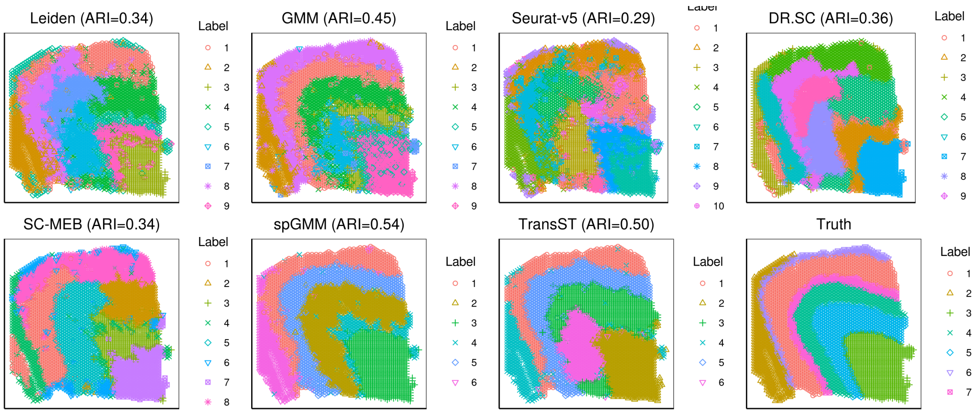

Fig. 9 Spatial heatmap for Sample 151674.

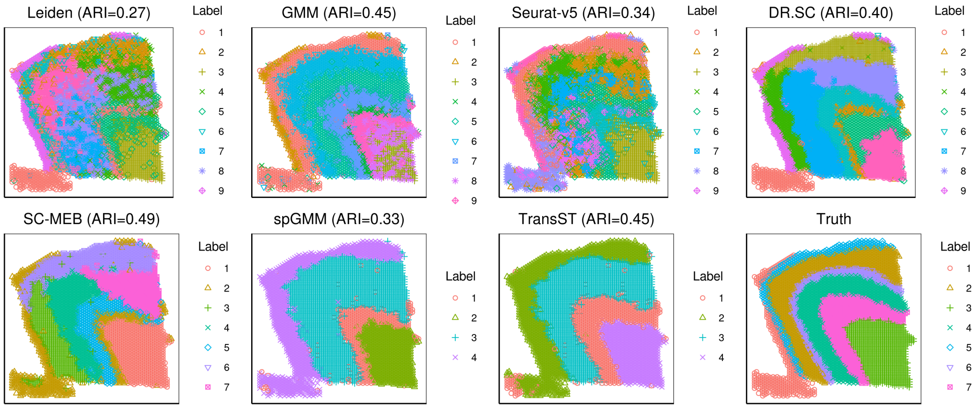

Fig. 10 Spatial heatmap for Sample 151675.

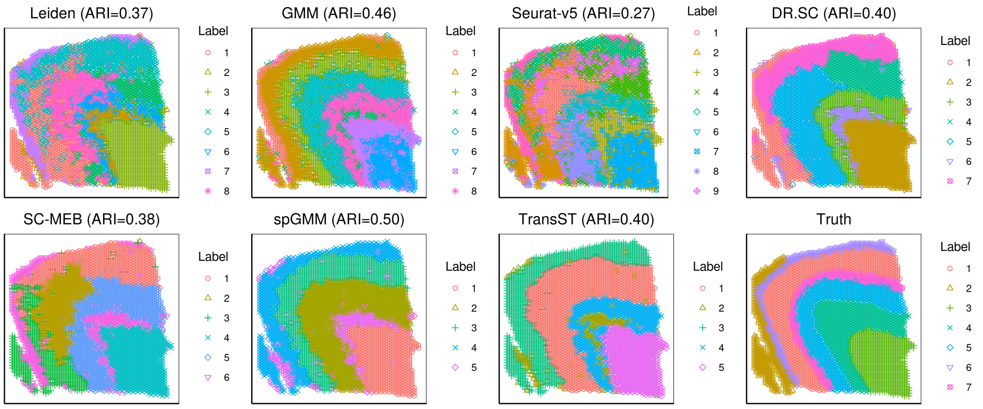

Fig. 11 Spatial heatmap for Sample 151676.

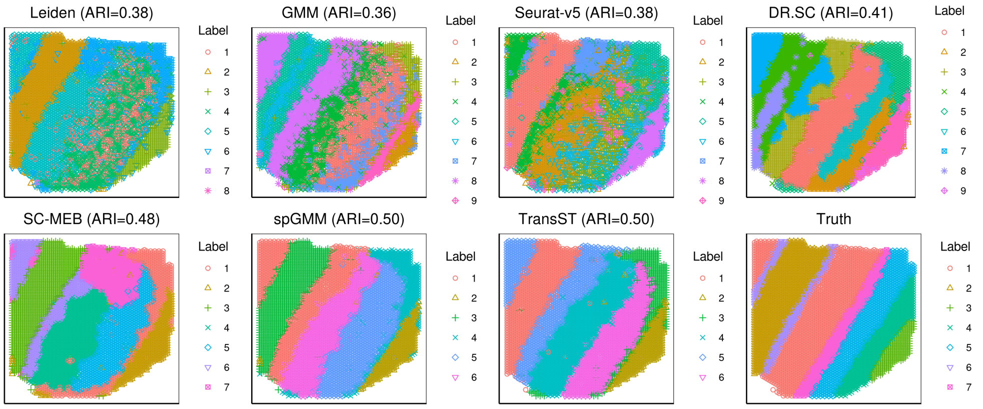

Fig. 12 Spatial heatmap for Sample 151676.

Table 1 Sample size n, number of genes p, and number of clusters in each sample.

	A1	B1	C1	D1	E1	F1	G2	H1
n	339	269	167	255	534	659	402	530
p	15045	15109	15557	15661	15701	14861	15258	15029
K	4	4	3	3	3	3	6	6

6 HER2 positive breast tumors

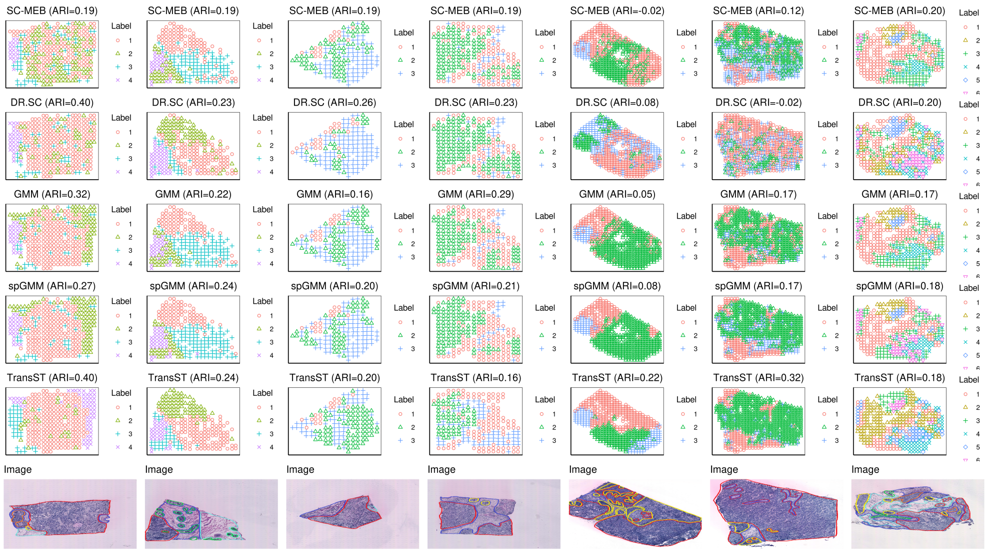

Fig. 13 Spatial heatmap for Samples A1, B1, C1, D1, E1, F1, and G2 (by column).

7 Mouse embryo

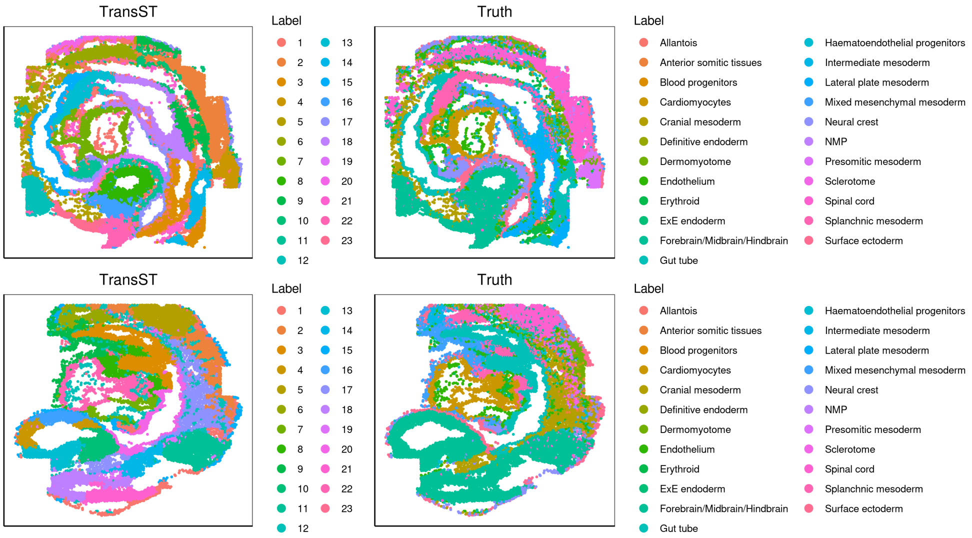

Fig. 14 Spatial heatmap for embryo 1 and embryo 2 (by row).

References

Besag, J.: On the statistical analysis of dirty pictures. Journal of the Royal Statistical Society Series B: Statistical Methodology **48**(3), 259–279 (1986)

Giraud, C.: Introduction to High-dimensional Statistics. CRC Press, ??? (2021)

Liu, W., Liao, X., Yang, Y., Lin, H., Yeong, J., Zhou, X., Shi, X., Liu, J.: Joint dimension reduction and clustering analysis of single-cell rna-seq and spatial transcriptomics data. Nucleic Acids Research **50**(12), 72–72 (2022)

Wang, L., Kim, Y., Li, R.: Calibrating non-convex penalized regression in ultra-high dimension. Annals of Statistics **41**(5), 2505 (2013)
